# Effects of Stroke Volume Maximization Before One-Lung Ventilation on Video-Assisted Thoracic Surgery: A Randomized Controlled Trial

**DOI:** 10.3390/diagnostics15111405

**Published:** 2025-05-31

**Authors:** Man-Ling Wang, Po-Ni Hsiao, Hsao-Hsun Hsu, Jin-Shing Chen, Ya-Jung Cheng

**Affiliations:** 1 Department of Anesthesiology, National Taiwan University Hospital, National Taiwan University College of Medicine, Taipei 100225, Taiwan; 2 Department of Surgery, National Taiwan University Hospital, National Taiwan University College of Medicine, Taipei 100225, Taiwan; 3 Medical Services Department, National Taiwan University Cancer Center, Taipei 100200, Taiwan

**Keywords:** fluid management, goal-directed fluid therapy (GDFT), stroke volume, thoracic surgery, one-lung ventilation

## Abstract

**Background/Objectives**: The use of goal-directed fluid therapy (GDFT) guided by stroke volume (SV) variation during thoracic surgery, particularly with one-lung ventilation (OLV) and protective ventilation strategies, is not well established. This study aimed to determine whether maximizing stroke volume (SV) before initiating one-lung ventilation (OLV) reduces the incidence of intraoperative hypotension requiring vasoactive agents during video-assisted thoracoscopic surgery (VATS). **Methods**: Sixty patients undergoing VATS were randomly assigned to an SVM group (*n* = 30) or a control group (*n* = 30). The SVM group received 6% hydroxyethyl starch before OLV to achieve and maintain an SV increase of less than 10%. The control group received no active fluid therapy before OLV positioning. Both groups received Ringer’s lactate solution intraoperatively based on baseline (control) or maximized (SVM) SV goals. The primary outcome was the use of vasoactive agents for hypotension. **Results**: Patients in the SVM group received significantly less Ringer’s lactate solution than controls (4.2 ± 2.4 vs. 6.1 ± 2.8 mL/kg/h, *p* = 0.005). While fewer patients in the SVM group required vasoactive agents (20% vs. 40%), the difference was not statistically significant (*p* = 0.091). IL-6 levels were significantly lower during OLV in the SVM group. **Conclusions**: Pre-OLV SVM was associated with reduced intraoperative crystalloid administration and attenuation of inflammatory response, with a non-significant trend toward lower vasopressor use. These findings suggest a potential benefit of SVM in thoracic surgery, though larger multicenter trials are needed to confirm clinical efficacy.

## 1. Introduction

A fluid diagnosis and treatment strategy for thoracic surgery has not been established. Liberal and excessive fluid administration may increase the risk of postoperative pulmonary complications [1,2,3]. However, a restrictive fluid therapy strategy may raise concerns regarding hypoperfusion and acute renal injury [4,5]. A recent retrospective study indicated that “moderate” intraoperative fluid management was associated with fewer postoperative pulmonary complications than restrictive or liberal management in patients undergoing thoracic surgery [6]. An important clinical question is the measurement of “moderate” for intraoperative fluid monitoring and administration.

In prospective studies in which fluid management was guided by stroke volume variation (SVV) during thoracic surgery, investigators observed benefits such as higher oxygenation with one-lung ventilation (OLV) [7,8], higher urine output [9], and fewer postoperative complications [8]. However, the efficacy of dynamic approaches or variation indexes to fluid therapy—including SVV and pulse pressure variation (PPV)—may be debatable because of protective ventilation strategies and unavoidable alterations in cardiopulmonary interaction during OLV [10,11]. Controversial results have also been reported regarding fluid management guided by SVV [8,12,13]. The use of variation indexes for goal-directed fluid therapy during thoracic surgery with OLV remains inconclusive.

The rationale behind dynamic indices, namely SVV and PPV, is based on intrathoracic pressure changes between inspiration and expiration during mechanical ventilation. This difference is mitigated with small tidal volumes during OLV. Currently, the literature supports the use of SVV only in patients ventilated with tidal volumes of more than 8 cc/kg [14]. In patients undergoing major non-cardiac and non-thoracic surgery, goal-directed hemodynamic therapy, including the administration of fluids, inotropes, and vasopressors guided by stroke volume (SV), mean arterial pressure, and cardiac index, improves outcomes [15]. Compared with SVV, SV is less affected by changes to ventilator settings. Therefore, SV monitoring may be more rational during thoracic surgery with OLV and lung protective ventilation strategy than SVV.

Atelectasis and re-expansion with OLV during thoracic surgery induce ischemia–reperfusion injury, including inflammatory [16,17] and oxidative responses [18,19]. Intraoperative fluid shifts may be augmented by lung manipulation during surgery, which may exaggerate inflammatory and oxidative injury. Because cardiac output (SV × heart rate) plays a crucial role in maintaining oxygenation with the unavoidable shunting that occurs during OLV, optimizing SV before OLV may result in fewer hypotensive episodes and smaller amounts of fluid administration after the initiation of OLV during surgery. Therefore, we developed a fluid management strategy to administer fluid and maximize SV (SVM) to achieve a blood volume at the plateau of the Frank–Starling curve by keeping the patient at a lower SV response level (i.e., an SV increase < 10% maintained for 20 min).

We hypothesized that SV was feasible for continuous monitoring in thoracic surgery with a protective ventilation strategy and OLV. The primary aim of this study was to determine whether SVM before OLV reduced hypotensive episodes during OLV. The secondary goal was to determine whether SVM before OLV was associated with reduced reperfusion injuries after the resumption of 2-lung ventilation. Decreased levels of serum biomarkers of oxidative, inflammatory, and organ injury may ensue.

## 2. Materials and Methods

### 2.1. Ethics

Ethical approval for this study (Ethical Committee No. 201605133RINB) was provided by the Research Ethics Committee of National Taiwan University Hospital, Taipei, Taiwan (Chairman Prof. Chun-Fang Tai) on 14 September 2016. All patients gave their written consent, and this study was registered at http://clinicaltrials.gov (accessed on 28 May 2025) with the identifier NCT03064880.

### 2.2. Patients

Patients who were scheduled for video-assisted thoracoscopic surgery and lung isolation between March 2017 and November 2017 were recruited. Patients were excluded if they had arrhythmias that intervened with the calculation of variation indexes; significant renal or hepatic disease that made manipulating fluid status difficult; or abnormal cardiopulmonary function, including heart failure beyond class II of the New York Heart Association Functional Classification, chronic obstructive pulmonary disease, or active coronary artery disease. Significant renal disease was defined as serum creatinine > 2.0 mg/dL or requiring dialysis. Significant hepatic disease was defined as Child–Pugh class B or C. Abnormal cardiopulmonary function included ejection fraction < 40%, FEV_1_ < 50% predicted, or DL_CO_ < 40% predicted. Active coronary artery disease was defined as angina within the past 6 months or recent myocardial infarction within the previous 3 months.

### 2.3. Anesthetic Management

General anesthesia was induced by intravenous administration of fentanyl (2 μg/kg), propofol (2 mg/kg), glycopyrrolate (0.2 mg), and rocuronium (1 mg/kg). After general anesthesia, lung isolation was conducted using either a double-lumen endobronchial catheter or a bronchial blocker. All patients were maintained with a sevoflurane–oxygen mixture, and the bispectral index was maintained between 40 and 60. The treatment protocols for respiratory and hemodynamic care were the same for each patient, including a tidal volume of 8 mL/kg for 2-lung ventilation and 5 mL/kg for one-lung ventilation with a positive end-expiratory pressure of 5 cmH_2_O. The fraction of inspired oxygen was set as low as possible, but SpO_2_ > 94% was maintained during surgery. The respiratory rate was adjusted to achieve end-tidal CO_2_ between 30 and 40 cmH_2_O. An arterial catheter was placed for hemodynamic monitoring, and the FloTrac™ system (Edwards Lifesciences Corp, Irvine, CA, USA) was used to derive hemodynamic indexes, including SV and cardiac index. The fluid management protocol was applied after anesthetic induction according to group assignment, as detailed in the following sections. A vasoactive agent such as ephedrine or norepinephrine was administered as an intravenous bolus if the change in mean arterial pressure was greater than 30% from the baseline.

### 2.4. Randomization and Group Assignments

We obtained written informed consent from all patients the day before surgery. Upon patients’ arrival at the operation theatre, randomization was performed using computer-generated random numbers in sequentially numbered, sealed envelopes. Their confidentiality was ensured by the research assistant, who was unaware of the patients’ characteristics and was not responsible for clinical work. The envelope was checked by a research anesthesiologist after confirming the patients’ cardiac index of at least 2.2 L/min/m^2^. Patients were randomly allocated to one of the study arms in a 1:1 ratio. The intervention of different fluid therapy was performed by the research anesthesiologist before patients changed position and before surgery. Participants, attending anesthesiologist, surgical staff, and postoperative care providers were blinded to the group allocation.

### 2.5. Fluid Management Protocols

After anesthetic induction, either (1) SV maximization (SVM) or (2) an SV maintenance (control) fluid management protocol was applied according to the group assignment:

(1) In the SVM group, fluid therapy with 250 mL of 6% hydroxyethyl starch (Voluven^®^ 6%, Fresenius Kabi Deutschland GmbH, Bad Homburg, Germany) was administered every 5 min until an SV change of less than 10% from previous value was maintained for 20 min. After pre-OLV SV maximization, the SV value was set as the fluid management goal for each patient in the group during surgery.

(2) In the control group, fluid therapy was guided by the baseline SV identified under 2-lung ventilation. After anesthetic induction and baseline SV determination, no proactive fluid bolus was administered prior to OLV. However, during the surgical procedure, intraoperative fluid titration using Ringer’s lactate solution was allowed to maintain the patient’s baseline SV level. SV was continuously monitored throughout surgery in both groups using the FloTrac™ system(Edwards Lifesciences Corp, Irvine, CA, USA) to guide ongoing fluid management and assess hemodynamic stability.

After the SV goal was identified for each patient, patients were placed in a lateral decubitus position, and OLV was initiated. During surgery, all patients were maintained with Ringer’s lactated solution at an initial infusion rate of 4 mL/kg/h, and it was adjusted to maintain the SV goal by the attending anesthesiologist. Arterial blood gas and 5 mL blood samples were collected at T1 (baseline, after completion of fluid therapy and before OLV), T2 (20 min after initiation of OLV), and T3 (20 min after resumption of 2-lung ventilation at the end of surgery).

### 2.6. Outcome Measurement

The primary outcome was intraoperative hypotension requiring the administration of vasoactive agents, which was set as a mean arterial pressure of less than 30% of the baseline in our institution [20]. Secondary outcomes included clinical outcomes and perioperative changes in serum biomarkers of inflammatory injury (interleukin 6, IL-6), oxidative injury (thiobarbituric acid-reactive substances, TBARS), lung epithelial injury (Clara cell secretory protein, CC16), pulmonary collectins (surfactant protein D, SPD), and acute kidney injury (neutrophil gelatinase–associated lipocalin, NGAL).

Intraoperative anesthetic management, including the use of vasoconstrictors, arterial blood gas, fluid therapy, blood loss, and urine flow rate, was collected from anesthetic records. Clinical data, including length of hospital stay, length of intensive care unit stay, and any cardiac, pulmonary, or other major organ complications, were collected from a chart review prior to discharge. Plasma levels of the biomarkers IL-6, TBARS, CC16, SPD, and NGAL at T1, T2, and T3 were measured and compared. Serum biomarker concentrations were measured using enzyme-linked immunosorbent assay kits.

### 2.7. Statistical Analysis

Calculations using a value of 0.05 and a statistical power of 0.8 revealed that a minimum of 24 patients in each group were required to detect a 50% reduction in intraoperative hypotension [20]. To compensate for an anticipated 20% dropout rate during the study, we recruited 30 patients to each group.

Statistical analysis was performed using Stata 14.2 (Stata Corp., College Station, TX, USA). Results were considered statistically significant for *p* values of <0.05. Findings are expressed as means ± standard deviation unless otherwise specified. Findings based on categorical data were tested using the chi-square test; otherwise, the parametric independent Student’s *t* test was used. All data derived from arterial blood gas and serum samples were analyzed using two-way analysis of variance (ANOVA) to evaluate the time effect, group effect, and interaction between the two factors.

## 3. Results

### 3.1. Demographic, Anesthetic, and Operative Results

**Figure 1:**
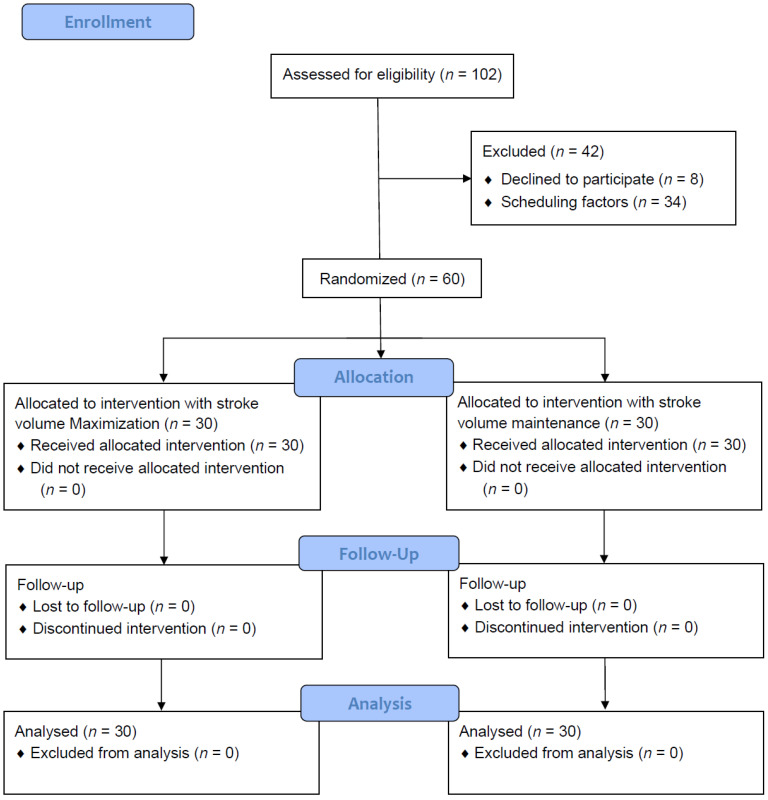
Patient recruitment scheme and CONSORT statement of this study.

Patients were recruited between 15 March 2017, and 29 November 2017. A total of 102 patients were screened for eligibility. The patient recruitment scheme is presented in Figure 1. As total of 60 patients were randomly allocated to one of the intervention groups (*n* = 30 in each group) and included in the analysis. The demographic data of the two groups are summarized in Table 1. The groups were comparable with respect to age, sex, body habitus, preoperative pulmonary function tests, pathological diagnosis, comorbidities, American Society of Anesthesiologists (ASA) classification, preoperative cardiac index, serum creatinine level, serum lactate level, and type of procedure performed. Anesthetic and operative results are presented in Table 2. Patients did not differ significantly between the groups in terms of induction time, operation time, or duration of one-lung ventilation. Patients in the SVM group received 338 ± 116 mL of 6% hydroxyethyl starch (Voluven^®^) for SV maximization. Patients in the control group received significantly more intraoperative Ringer’s lactated solution (6.1 ± 2.8 vs. 4.2 ± 2.4 mL/kg/h) than the SVM group. The urine flow rate was similar between the two groups. The OLV and surgical duration did not differ between the groups. No pneumonectomy or conversion to thoracotomy was performed. Overall, 16 patients underwent lobectomy, and one segmentectomy was performed in the SVM group. A total of 20 patients underwent lobectomy in the control group. All remaining patients underwent either wedge resections or no pulmonary resection.

### 3.2. Primary Outcome

Fewer patients in the SVM group than in the control group experienced severe hypotension that required intraoperative vasoactive agents but did not meet statistical significance (20% vs. 40%, *p* = 0.091). In the SVM group, five patients required ephedrine, and one required norepinephrine during surgery, whereas in the control group, ten patients required ephedrine, and two required norepinephrine. The relative risk of intraoperative hypotension requiring vasopressors was reduced by 50% (95% CI −15.7% to 78.4%). The absolute risk reduction was 20% (95% CI −2.6% to 42.6%).

### 3.3. Secondary Outcome

No perioperative mortality, pneumonia, or dysrhythmia occurred in either group. Moreover, eight patients developed prolonged air leakage for longer than five days in each group. The length of hospital stay did not differ between the groups (4.1 vs. 4.1 d, *p* = 0.413).

The serum biomarker levels are presented in Table 3. IL-6 decreased significantly after initiation of OLV in the SVM group (mean: 3.7–1.7 pg/mL) but not in the control group (mean: 6.0–9.6 pg/mL). IL-6 increased significantly after resuming 2-lung ventilation at the end of surgery in both groups (mean: 32.8 pg/mL in the SVM group and 38.3 pg/mL in the control group). The levels of CC16, TBARS, NGAL, and SPD did not change significantly between groups at different time points.

A two-way ANOVA was performed to analyze the effect of time and group on the serum levels of biomarkers. A two-way ANOVA revealed a statistically significant interaction between the effects of time and group on IL-6 (*p* < 0.001). Simple main effects analysis showed that both time and group had statistically significant effects on IL-6 (both *p* < 0.001). We did not observe a statistically significant effect of time and group on CC16, TBARS, NGAL, or SPD.

## 4. Discussion

Pre-OLV stroke volume maximization (SVM) showed promise in reducing intraoperative fluid requirements and inflammatory markers, particularly IL-6, in patients undergoing thoracoscopic surgery. Although the reduction in vasopressor use in the SVM group did not achieve statistical significance, this finding is clinically relevant and warrants further exploration.

Although optimal cardiac output is essential for oxygenation during OLV with unavoidable shunting, no guide exists for optimizing cardiac output before OLV initiation. Because cardiac output can be affected by multiple factors, including anesthetic depth, fluid status, and preoperative cardiovascular diseases, we presented a protocol for maximizing SV after achieving optimal anesthetic status before the operation. SV maximization, unlike other fluid management strategies that use SVV or PPV, is less affected by changes in position and ventilation settings. Cardiac output may not be increased during SVM in patients with different myocardial contractility or heart rate responses; however, pre-OLV SVM is a reasonable approach for fluid management through individualized optimization of anesthesia administration and fluid therapy.

The reason for choosing SV rather than SVV was that the use of a lung protective ventilation strategy involved lower tidal volume compared with conventional mechanical ventilation. Dynamic preload indices, including SVV and PPV, are based on variation in intrathoracic pressure from cardiopulmonary interactions and require a larger tidal volume to derive predictions of preload responsiveness [21,22]. This was also acknowledged during OLV and thoracic surgery [23,24]. The use of SV as the goal for fluid therapy may be less affected by a lung protective ventilation strategy.

Our results indicate that pre-OLV SVM is beneficial for attenuating intraoperative fluctuations of blood pressure, with comparable kidney functions such as urine flow rate and NGAL in patients with normal renal function. Intraoperative hypotension can result in additional fluid administration and blood pressure fluctuations requiring vasoactive agents. With the maintenance instead of maximization of SV, more hypotensive episodes occurred, and vasopressors were used more frequently, despite a significantly higher average intraoperative infusion rate with crystalloids. Fluid optimization using the SVM technique before surgery reduced the required amount of intraoperative fluid administration and resulted in a hemodynamically stable status.

The timing of fluid therapy was the key difference between the two groups. The total fluid administration during the operation was similar between the groups (813.7 ± 299.5 mL in the SVM group vs. 720 ± 337.2 mL in the control group). There was a misconception that goal-directed fluid therapy, especially SV manipulation, may lead to excessive fluid compared with the conventional approach [25]. Our study indicates that SVM before OLV is associated with smaller intraoperative fluid administration and better hemodynamic stability during surgery. The retention of fluid administered during surgery can result in postoperative tissue edema. Even though the total volume was similar between the fluid therapies, the timing of administration may have had an impact on outcomes and recovery after surgery.

Our results also indicate that pre-OLV SVM attenuates the increase in IL-6 levels after resuming 2-lung ventilation but not the increase in levels of TBARS. IL-6 was selected as a representative biomarker of systemic inflammation due to its prompt and transient induction in response to tissue injury and surgical trauma. Its clinical relevance in predicting surgical and critical care outcomes is supported in the literature [26]. Among all evaluated biomarkers, only IL-6 demonstrated a statistically significant interaction over time between the groups. Changes in other biomarkers, including CC16, TBARS, NGAL, and SPD, did not reach statistical significance and should be interpreted with caution. The thoracoscopic technique has been significantly improved, with shorter OLV duration and fewer thoracoscopic ports. According to our results, the TBARS level, a marker of oxidative injury, did not exhibit significant differences between the time points. However, the CC16 level, which was significantly increased during OLV and highest at T3, indicated unavoidable epithelial injury resulting from surgical manipulation. Although serum CC16 is a sensitive biomarker for increased leakiness of the lung epithelial barrier, SPD levels did not change significantly, which contrasts with a previous study of patients with acute lung injury [27].

Compared with the changes in the CC16 level, levels of IL-6, but not CC16, significantly decreased during OLV in the SVM group after the initiation of OLV. These results may indicate that CC16 is mostly related to the injury of pulmonary epithelial cells; however, plasma IL-6 may have been highly associated with reperfusion injury after resuming 2-lung ventilation after OLV.

We acknowledge the concerns surrounding the use of hydroxyethyl starch (HES) for volume expansion. At the time this study was designed, HES was commonly employed in our institution for intraoperative fluid management. No adverse renal events were observed in the enrolled patient cohort. Nevertheless, we recognize the ongoing safety concerns reported in the literature, and we agree that future studies should consider alternative volume expanders with more favorable risk profiles.

A post hoc power analysis indicated that the study had approximately 40% power to detect the observed difference in vasopressor use, suggesting the study may have been underpowered for the primary outcome. Future research with larger sample sizes and multicenter collaboration is necessary to validate the hemodynamic and clinical benefits of the SVM protocol.

Heterogeneity in surgical procedures (lobectomy, wedge resection, segmentectomy, and mediastinal surgery) may also have influenced intraoperative hemodynamic responses and biomarker dynamics. This factor should be considered when interpreting the results and may warrant stratification in future studies. Additionally, although this study only included thoracoscopic procedures, we do not consider this a limitation. Instead, we suggest that future research explore comparisons between stroke volume maximization in VATS and thoracotomy cases to evaluate its broader applicability.

### Limitations

This study has several limitations that may affect the interpretation and generalizability of the findings. First, the single-center design and relatively small sample size may limit the broader applicability of the results and contribute to potential bias due to institutional standards of care. Second, SV was derived from the FloTrac™ system(Edwards Lifesciences Corp, Irvine, CA, USA), which precluded patients with arrhythmias. SV was calculated using biometric data derived from patient weight and height rather than directly measured cardiac output, which may have introduced bias. Furthermore, only thoracoscopic procedures were conducted. Intraoperative hemodynamics and fluid requirements during thoracotomy may be different. The pattern of serum biomarker changes may be a concern among different timings. In our previous investigation, the highest levels for injurious biomarkers were noted after resuming 2-lung ventilation rather than at postoperative day 1 [28].

## 5. Conclusions

Pre-OLV stroke volume maximization (SVM) provides a structured approach to intraoperative fluid management in thoracoscopic surgery with one-lung ventilation. Our findings indicate that SVM is feasible, reduces intraoperative crystalloid requirements, and may attenuate inflammatory responses, as reflected by lower IL-6 levels. While there was a trend toward reduced hypotension requiring vasopressors, this did not reach statistical significance. Given the single-center design and limited sample size, these findings should be interpreted as preliminary and hypothesis-generating. Future larger, multicenter randomized trials are necessary to confirm the potential benefits of SVM on hemodynamic stability and postoperative outcomes.

## Figures and Tables

**Table 1:** Patient characteristics.

Variable	SV Maximization (*n* = 30)	SV Maintenance (*n* = 30)	*p* Value
Age, yr	57.9 ± 9	55 ± 8.2	0.086
Female, *n* (%)	19 (63)	23 (77)	1
Height, cm	160.2 ± 8.1	158.8 ± 23.2	0.412
Weight, kg	62.7 ± 11.7	64.4 ± 13.6	0.641
Preoperative cardiac index, L/min/m^2^	2.6 ± 0.5	2.7 ± 0.5	0.279
Preoperative creatinine, mg/dL	0.81 ± 0.15	0.76 ± 0.16	0.108
Preoperative lactate, mg/dL	1.16 ± 0.44	0.94 ± 0.34	0.064
Preoperative pulmonary function test			
FVC (%)	113.2 ± 15.5	108.3 ± 9.9	0.176
FEV_1_ (%)	111.9 ± 16.5	107 ± 10	0.403
History of smoking, *n*	1	0	0.336
Coexisting disease			
Hypertension, *n*	8	5	0.3
Diabetes mellitus, *n*	3	5	1
Asthma, *n*	1	1	1

Abbreviations: FVC, forced vital capacity; FEV_1_, forced expiratory volume in 1 s; SV, stroke volume.

**Table 2:** Operative data.

Variable		SV Maximization (*n* = 30)	SV Maintenance (*n* = 30)	*p* Value
Lung separation device, *n*	DLT	8	6	1
	Left BB	11	13	1
	Right BB	11	11	1
Duration, min	Induction	16.6 ± 7.8	15.6 ± 9.2	0.384
	Anesthesia	175.8 ± 52.7	181.9 ± 71.6	0.935
	Operation	122.1 ± 45.3	119.1 ± 44.2	0.399
	One-lung ventilation	105 ± 43	104 ± 41	0.872
Operative method, *n*				0.748
	Lobectomy	17	21	
	Segmentectomy	1	1	
	Wedge resection	9	6	
	Mediastinal surgery	3	2	
Vasopressor	Use of ephedrine or norepinephrine, *n* (%)	6 (20)	12 (40)	0.091
Ventilation	Highest ETCO_2_, mmHg	42.3 ± 4.2	44.4 ± 4.3	0.07
	Lowest SpO_2_ (%)	95 ± 6	97 ± 3	0.145
Intravenous fluids	Voluven^®^, mL	338 ± 116	0	NA
	Crystalloid rate, mL/kg/h	4.2 ± 2.4	6.1 ± 2.8	0.005
Urine flow rate, mL/kg/h		1.63 ± 1.25	1.66 ± 2.67	0.062

Abbreviations: BB, bronchial blocker; DLT, double-lumen endo-bronchial tube; ETCO_2_, end-tidal carbon dioxide; SV, stroke volume.

**Table 3:** Changes in perioperative plasma levels of one-lung ventilation-induced injurious biomarkers.

Variable	SV Maximization (*n* = 30)			SV Maintenance (*n* = 30)			Difference by Two-Way ANOVA (*p* Value)		
Plasma level of biomarkers	T1	T2	T3	T1	T2	T3	Time effect	Group effect	Time and group effect
CC-16	10.4 ± 5.8	13.5 ± 7.2	21.5 ± 14.9	9.6 ± 5.8	13.3 ± 7.2	19.9 ± 14.9	<0.001	0.535	0.930
IL-6	3.7 ± 7.5	1.7 ± 3.2	32.8 ± 37.7	6.0 ± 10.4	9.6 ± 16.3	38.3 ± 46.3	<0.001	<0.001	<0.001
TBARS	11 ± 4.9	11.7 ± 7.9	15.0 ± 11.3	10.6 ± 9.0	11.5 ± 9.3	10.9 ± 10.0	0.237	0.133	0.242
NGAL	132.2 ± 70.1	118.3 ± 50.6	162.2 ± 114.8	142.3 ± 53.3	146.0 ± 56.3	163.0 ± 72.8	0.252	0.058	0.566
SPD	100.1 ± 74.8	91.8 ± 69.8	85.8 ± 63.2	81.7 ± 63.9	80.5 ± 66.6	76.2 ± 61.1	0.723	0.310	0.939

Abbreviations: CC-16, Clara cell secretory protein; IL-6, interleukin 6; NGAL, neutrophil gelatinase–associated lipocalin; SPD, surfactant protein D; SV, stroke volume; TBARS, thiobarbituric acid-reactive substances.

## Data Availability

The dataset is available upon request from the authors.

## Abbreviations

The following abbreviations are used in this manuscript:

ANOVA	Analysis of variance
ASA	American Society of Anesthesiologists
BB	Bronchial blocker
CC16	Clara cell secretory protein
DLT	Double-lumen endo-bronchial tube
ETCO_2_	End-tidal carbon dioxide
FEV_1_	Forced expiratory volume in 1 s
FVC	Forced vital capacity
GDFT	Goal-directed fluid therapy
IL-6	Interleukin 6
NGAL	Neutrophil gelatinase–associated lipocalin
OLV	One-lung ventilation
PPV	Pulse pressure variation
SPD	Surfactant protein D
SV	Stroke volume
SVM	Stroke volume maximization
SVV	Stroke volume variation
TBARS	Thiobarbituric acid-reactive substances
VATS	Video-assisted thoracoscopic surgery
